# Long-stay patients in pediatric intensive care unit: Diagnostic-specific definition and predictors

**DOI:** 10.1371/journal.pone.0223369

**Published:** 2019-10-02

**Authors:** Angelo Polito, Christophe Combescure, Yann Levy-Jamet, Peter Rimensberger, on behalf of the Swiss Society of Intensive Care Medicine

**Affiliations:** 1 Pediatric and Neonatal Intensive Care Unit, Department of Pediatrics, University Hospital of Geneva, Geneva, Switzerland; 2 Division of Clinical Epidemiology, Faculty of Medicine, University of Geneva, and Geneva University Hospitals, Geneva, Switzerland; Wayne State University, UNITED STATES

## Abstract

**Aims:**

To stipulate a new definition for long-stay patients (LSPs) in pediatric intensive care unit (PICU). We defined LSPs as the 10% of patients with the longest PICU length-of-stay (LOS) for each age and diagnostic group. To assess whether the thresholds (days of PICU stay) for the definition of LSPs in PICU significantly differ among diagnostic and age categories. To determine whether independent associations exist between patients’ characteristics at admission and LSPs diagnosis in pre-specified diagnostic and age groups.

**Methods:**

This was a multicenter retrospective cohort study including all PICUs in Switzerland. Multivariable regression analysis was used to seek for association between patients’ variables at admission and LSPs

**Results:**

We included 22,284 patients with a median (IQR) age of 12 (1–84) months. Significantly different thresholds across diagnostic and age subgroups are identified. Readmission to PICU, higher PIM2 and NEMS (a score used to quantify nursing workload at intensive care unit level) at admission were associated with higher likelihood of becoming LSPs.

**Conclusions:**

Our results showed a significantly different definitions of LSPs for specific diagnoses and age categories. Readmission to PICU and higher acuity at admission are associated with longer PICU length-of-stay in the majority of diagnostic groups. A more personalized definition of LSPs in children based on actual patients’ characteristics should probably be used in an effort to optimize care and reduce costs.

## Introduction

An increased number of patients survive a critical illness or surgical procedure [1]. As a consequence a unique group of patients with extended length-of-stay (LOS) has emerged [2]. These ‘long-stay patients’ (LSPs) are left with increased morbidity and higher mortality rates [3]. These patients also consume more resources than patients with shorter LOS [4]. At present, a standard definition of LSPs is not available. Actual definitions of LSPs mostly rely on fixed LOS cutoff points (e.g. 14 days) alone or in combination with clinical features such as ongoing technology dependence, a proportion of patients with the longest LOS or the visual analysis of the ‘tail’ of the LOS distribution [5,6]. Nevertheless none of these definitions take into account diagnosis and age differences of PICU patients. A single cut-off point applied to the entire PICU population regardless of patients’ primary diagnosis might effectively fall in the ‘tail’ of the LOS distribution curve for one diagnostic category but the same cut-off might represent the median value of LOS for another group of patients. Our aim was to stipulate a new definition for LSPs in PICU. A LSPs definition based on a specific proportion of patients (i.e. the 10% with the longest stays) has the theoretical advantage of systematically identifying outliers of the LOS distribution curve of the population of patients in exam [5]. We hypothesized that the application of this definition of LSPs to specific diagnostic categories would probably identify significantly different thresholds. The identification of LSPs for individual diagnosis might be used for the development of diagnostic-specific clinical pathways, to reduce variations in care and make timely family support decisions. [6].We also sought to determine independent associations between patients’ characteristics at admission and LSPs diagnosis in pre-specified diagnostic groups.

## Materials and methods

### Study design

This retrospective study analyzed data from the Minimal Intensive Care Unit Dataset (MDSi) of the Swiss Society of Intensive Care Medicine that systematically collects information on all pediatric admissions to all PICU in Switzerland. The study was approved by the Ethical Commission of Northwestern Switzerland (EKNZ UBE-15/47) and the Scientific Committee of the Swiss Society for Intensive Care Medicine with a waiver for informed consent.

### Patients

The study population is represented by all children aged 16 years or younger admitted to any PICU in Switzerland between January 1, 2012, and December 31, 2017. Data from preterm neonates in Switzerland are collected on a separate national neonatal database [7] and therefore were not included in the analysis. The aim of this study was to evaluate the impact of diagnosis at admission (defined as the diagnosis that best describes the reason for admission to the PICU) on the definition of LSPs; therefore children who could not be identified with one primary admission diagnosis were excluded. For patients transferred to/coming from other PICUs (2078 cases, 8%) the exact PICU LOS could not be established (a new health record number is reassigned to patients who are admitted to another unit), therefore they were also excluded from the study.

### Data

All 8 tertiary PICUs in Switzerland collect data to the MDSi using a standard coding for admissions. Since 2012, the pediatric MDSi uses the ANZPIC Registry diagnostic codes to classify children into the following primary admission categories: cardiac (medical and surgical), cardiac or respiratory arrest, trauma, neurology, oncology, respiratory, sepsis with or without septic shock, miscellaneous [8]. Further variables extracted from the pediatric MDSi were: year of admission, gender, PICU LOS, readmission within 48 hours from last PICU admission, age and diagnosis of admitted children, chromosome anomaly, pneumonitis, major airway anomalies, acute renal failure, acute liver insufficiency, chronic lung disease, single-ventricle physiology, bone marrow transplant, Pediatric Index of Mortality (PIM) 2 and nursing manpower scale (NEMS) score at admission. The PIM2 score predicts individual patient outcomes to determine aggregate mortality rates of PICUs or group of patients according to physiologic data available at admission [9]. The NEMS is frequently used to quantify, evaluate and allocate nursing workload at intensive care unit level [10]. It scores 9 representative items of treatments (e.g. vasoactive medications, mechanical ventilator support) and it is calculated at the end of each nursing shift.

### Statistical methods

Data are summarized as frequencies and percentages for categorical variables and as median with interquartile range (25^th^-75^th^ percentile) for continuous variables.

#### Application of the LSPs definition to specific diagnostic and age categories

Diagnosis and age at admission were used to create subgroups of patients. Depending on the primary admission category, patients were divided into one of these major categories: cardiac, cardiorespiratory arrest, injury, neurological, oncology, respiratory, sepsis and miscellaneous. Patients’ age was categorized according to 3 main groups: neonates (<1 months), infants (1–11 months) and pediatric (1–16 years). For each diagnostic and age category, LSPs were defined as the 10% of patients with the longest PICU LOS [11]. Mann Whitney or Kruskal Wallis tests were used as appropriate to evaluate whether significantly different median LOS exist according to patients’ characteristics (gender, age and diagnostic category at admission, readmission within 48 hours, medical/surgical indication and death). Chi-square tests were used to assess whether significantly different thresholds for LSPs definition exist among age and diagnostic categories.

#### Association between patients’ characteristics at admission and LSPs diagnosis

Univariate and multivariable logistic regression models were used to determine adjusted relationships between patients’ characteristics and LSPs. The factors investigated included the following variables: age, sex, PIM2 score, first measured NEMS, chromosome anomaly, pneumonitis, major airway anomalies, acute renal failure, acute liver insufficiency, chronic lung disease, single-ventricle physiology, and bone marrow transplant. Covariates with a value of p < 0.05 by univariate analysis were included in multivariable analyses. The probability to develop the end-point for PIM2 and NEMS was not linear; thus, these variables were analyzed by quintiles of distribution. These analyses were conducted by category of diagnosis. All statistical tests were two-sided and the significance level was 0.05. All analyses were performed using R statistical software version 3.5.0 (The R Foundation for Statistical Computing).

## Results

A total of 22,284 patients were analyzed. Demographic characteristics for these patients are summarized in Table 1. Main diagnostic categories were represented by ‘miscellaneous’ (28.5%) followed by patients with respiratory indications (27.4%). Most frequent medical diagnosis found among ‘miscellaneous’ patients were represented by gastrointestinal issues/bowel obstruction (8%), patients needing PICU surveillance after invasive procedures (4%) and patients with decompensated diabetes (3%). Mortality rate for the entire cohort was 2%.

**Table 1:** Demographic characteristics for the whole patient population.

Variable	
Male, n (%)	12.793 (57.2)
Female, n %	9.589 (42.8)
Age at admission, months	12 (1–84)
LOS, days	1.44 (0.8–3.6)
PIM 2 score	1.4 (0.6–3.2)
Readmission within 48h, n (%)	466 (2.1)
NEMS at admission	18 (15–27)
Diagnostic group, n (%)	
CARDIAC	4052 (18.2)
Cardiac surgical	1802 (45.2)
Cardiac medical	2187 (54.8)
C.R. ARREST	106 (0.5)
INJURY	1786 (8.0)
MISCELLANEOUS	6348 (28.5)
NEUROLOGICAL	3151 (14.1)
ONCOLOGY	350 (1.6)
RESPIRATORY	6104 (27.4)
SEPSIS	387 (1.7)
Mortality, n (%)	445 (2.0)

Continuous variables are presented as median (IQR); Total patients = 22.284 LOS: length-of stay; PIM 2: Pediatric Index of Mortality 2; NEMS: Nine equivalent of nursing man power scale score; C.R. ARREST: cardiorespiratory arrest.

Distribution of LOS according to demographic characteristics is summarized in Table 2. Median LOS significantly decreased from 1.7 in 2012 to 1.3 days in 2017 (p < .0001). Gender, age at admission and PICU outcome significantly affected median LOS as male, younger patients and those who survived PICU admission had longer stay. Cardiac surgical and septic patients stayed longer with a median LOS of 3 days, compared to other diagnostic groups. Among patients who died, the majority expired within the first 8 days of admission (70.6%) while only 10.3% of patients died after 28 days. Nonetheless case fatality increased each day for patients who remained in PICU (6%, 10% and 15% at 8, 14 and 28 days respectively). A total of 2089 (9.3%) children were defined LSPs. Significantly different cut-off points (days of PICU stay) across diagnostic and age subgroups are identified.

**Table 2:** Distribution of LOS according to demographic characteristics.

Variable	Median LOS (IQR)	P value
Female	1.3 (0.8–3.3)	.006
Male	1.5 (0.8–3.7)	
Age at admission, y		< .0001
< 1 m	2.8 (1.2–5.7)	
1–11 m	1.7 (0.9–4.3)	
1–16 y	1.0 (0.7–2.2)	
Readmission within 48h		< .0001
Yes	2.1 (0.9–4.6)	
No	1.4 (0.8–3.5)	
Diagnostic group		< .0001
CARDIAC	2.4 (1.1–5.2)	
Cardiac surgical§	3.0 (1.3–6.3)	
Cardiac medical§	2.1 (1.1–4.3)	
C.R. ARREST	1.9 (0.7–3.8)	
INJURY	0.9 (0.6–1.7)	
MISCELLANEOUS	1.0 (0.7–2.6)	
NEUROLOGICAL	1.1 (0.7–3.2)	
ONCOLOGY	0.9 (0.3–1.9)	
RESPIRATORY	1.7 (0.7–4.0)	
SEPSIS	3.0 (1.4–6.1)	
Mortality		< .0001
Yes	1.0 (0.7–2.8)	
No	1.7 (0.8–4.0)	

Total patients = 22.284; LOS: length-of stay; C.R. ARREST: cardiorespiratory arrest.

§ p < .0001 for comparison between cardiac surgical and medical patients

**Fig 1:**
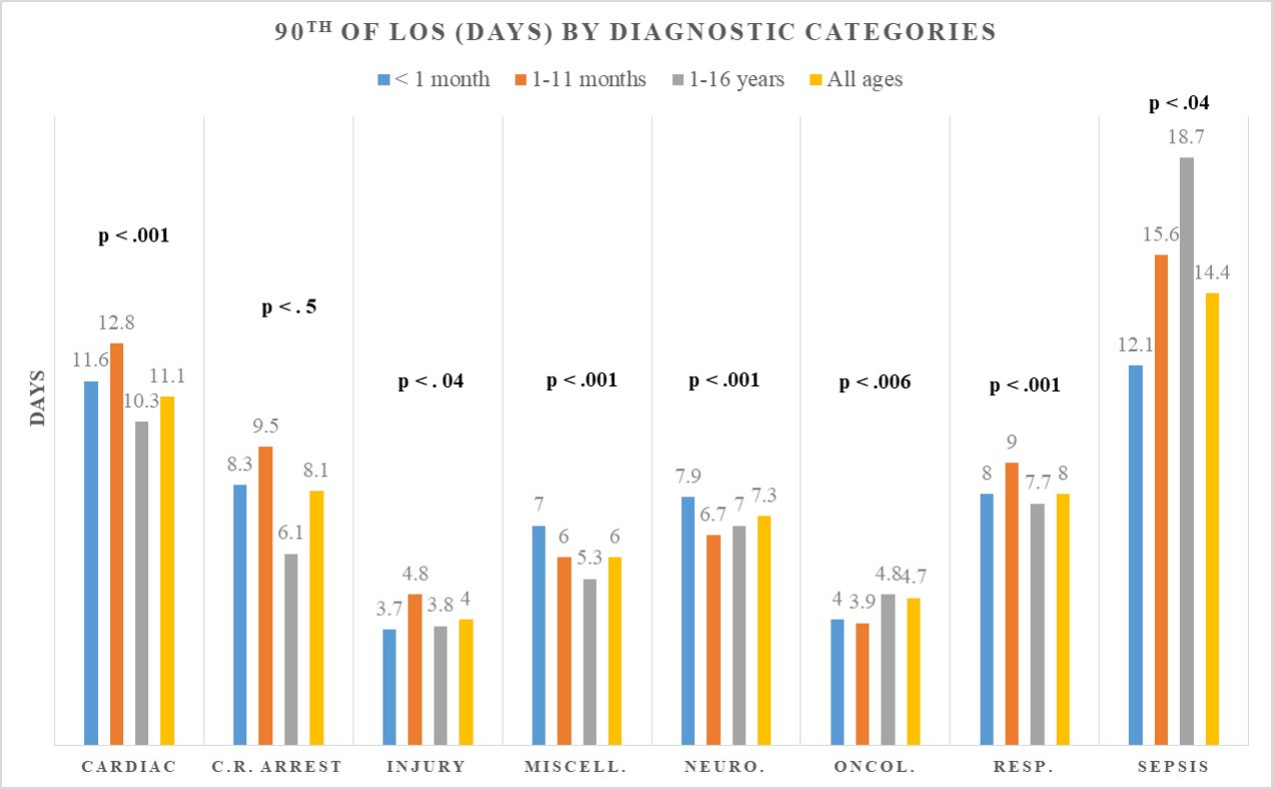
Differences in LSPs definition as identified by 90^th^ percentile of LOS distribution by diagnostic categories. LOS = length-of stay; C.R. ARREST: cardiorespiratory arrest; diag.: diagnoses.

Significantly different thresholds for the definition of LSPs were identified for each age subgroup within all diagnostic categories, except for cardiorespiratory arrest patients. (Fig 1).

**Fig 2:**
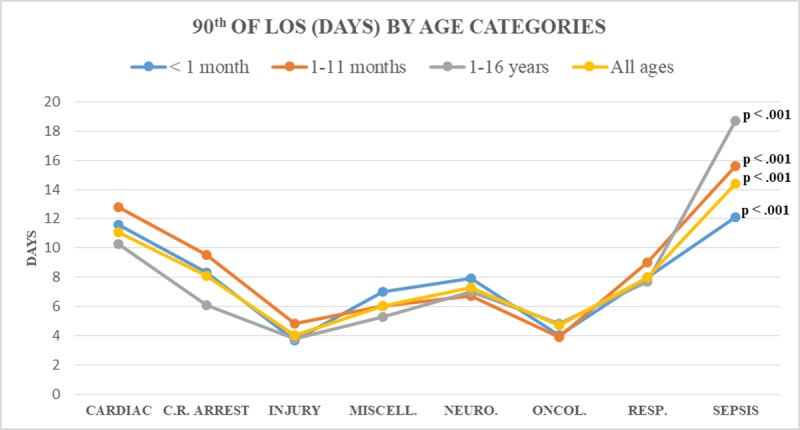
Differences in LSPs definition as identified by 90^th^ percentile of LOS distribution by age categories.

Similarly, within the same age subgroup, significantly different thresholds for LSPs definitions were identified across diagnostic categories (Fig 2). For instance, the 90^th^ percentile for septic and injured infants (1-11months) was 15.6 and 4.8 days, respectively.

As a consequence of their small group size, multivariate analyses were not performed for oncologic and septic patients. Patients’ characteristics associated with greater odds of becoming LSPs for all diagnostic categories are shown in Table 3. Higher NEMS at admission (40–51) was associated with greater odds of being defined as LSPs in all but neurological patients. Likewise higher probability of death (>20%) as predicted by PIM2 score was independently associated with becoming LSPs in all but injured patients. The presence of pneumonia and major airway anomalies were both independently associated with being LSPs in patients with respiratory indications. Given the very low frequencies of several patients’ characteristics at admission, the association between certain variables (i.e. genetic syndrome, chronic lung disease, tracheostomy, single-ventricle physiology) with the main outcome has not been explored.

**Table 3:** Multivariate associations between patients’ characteristics and LSPs definition.

	CARDIAC		INJURY		MISCELLANEOUS		NEUROLOGICAL		RESPIRATORY	
	OR (95%CI)	*P*	OR (95%CI)	*P*	OR (95%CI)	*P*	OR (95%CI)	*P*	OR (95%CI)	*P*
**Age**										
< 1 m	referent		referent		referent		referent		referent	
1–11 m	0.72 (0.56–0.93)	0.01	0.49 (0.15–1.56)	0.05	0.48 (0.38–0.60)	<0.001	0.62 (0.42–0.89)	0.0107	2.25 (1.78–2.84)	<0.001
1–16 y	0.26 (0.19–0.34)	<0.001	0.30 (0.12–0.79)	0.3	0.25 (0.20–0.30)	<0.001	0.27 (0.20–0.37)	<0.001	1.09 (0.87–1.36)	0.5
**Readmission**	1.43 (0.73–2.79)	0.3	1.27 (0.40–4.10)	0.7	2.29 (1.40–3.75)	<0.001	2.20 (1.22–3.97)	0.01	2.42 (1.43–4.10)	0.001
**NEMS**										
0–9	referent		referent		referent		referent		referent	
10–19	1.31 (0.70–2.10)	0.3	2.07 (0.84–5.14)	0.1	1.39 (1.02–1.91)	0.04	1.25 (0.76–2.06)	0.4	0.84 (0.55–1.29)	0.4
20–29	1.48 (0.75–2.18)	0.1	10.6 (4.41–25.34)	<0.001	2.7 (1.95–3.67)	<0.001	1.35 (0.82–2.23)	0.7	1.38 (0.91–2.08)	0.1
30–39	1.21 (0.71–2.07)	0.5	36.1 (14.44–90.26)	<0.001	4.4 (3.11–6.38)	<0.001	2.03 (1.18–3.47)	0.01	2.09 (1.36–3.21)	<0.001
40–51	1.87 (1.04–3.39)	0.04	54.5 (17.9–184.6)	<0.001	13.3 (6.75–26.08)	<0.001	1.89 (0.87–4.10)	0.1	4.09 (1.86–8.97)	<0.001
**PIM2 (%)**										
0–1.9	referent		referent		referent		referent	—	referent	
2–4.9	1.64 (1.24–2.16)	<0.001	1.31 (0.82–2.11)	0.3	1.67 (1.35–2.07)	<0.001	2.20 (1.55–3.13)	<0.001	1.76 (1.37–2.26)	<0.001
5–9.9	2.98 (2.09–4.26)	<0.001	1.67 (0.97–2.88)	0.07	2.52 (1.88–3.38)	<0.001	2.26 (1.52–3.36)	<0.001	2.18 (1.66–2.85)	<0.001
10–19.9	4.61 (3.10–6.85)	<0.001	3.00 (1.40–6.42)	0.005	3.03 (2.08–4.43)	<0.001	3.52 (2.16–5.73)	<0.001	5.75 (4.33–7.65)	<0.001
20–100	5.90 (3.86–9.03)	<0.001	1.67 (0.86–4.72)	0.1	2.91 (1.80–4.72)	<0.001	4.96 (3.23–7.61)	<0.001	4.95 (3.17–7.73)	<0.001
Pneumonia									2.78 (1.42–5.42)	.003
M. air. an.									1.67 (1.19–2.35)	.003

OR indicates odds ratio; CI, confidence interval;M.air.an., major airways anomalies

## Discussion

Our results showed that significantly different thresholds for LSPs definition exist across different diagnostic and age groups. Similar conclusions have been reached in both the adult and pediatric populations [3,12].

Families facing longer hospitalizations are less informed than those experiencing shorter hospitalizations [13] and lack crucial information that is essential to thoughtful decision making [14]. In another study, families of patients receiving mechanical ventilation for prolonged periods frequently misunderstand the need for a tracheotomy as a move toward recovery, rather than a sign of protracted frailty [15]. The unselective use of predefined thresholds for LSPs definition (i.e. 14 days) might inappropriately (either too early or too late) classify patients as LSPs. Adapting LSPs definition to the patients’ characteristics would facilitate effective clinician-family communication and help to reshape the goals of care and make meaningful medical decisions with families. For instance, oncologic patients would be considered as LPS compared to other oncologic patients if LOS exceeds 5 days, whereas septic patients would be regarded as LPSs if their LOS exceeds 15 days. Such stratification might help to identify patients with uncommon/exceptional LOS for each diagnosis, prompt effective communication with families and possibly develop diagnostic-specific critical care pathways [16].

The fast-growing LSPs population also use a disproportionate amount of health-care resources [17]. Long-stay patients adversely affect profit margin in PICU when reimbursement is based on diagnosis-related group coding [18]. Focusing on LPSs group especially with regard to specific diagnostic subgroups might help refine reimbursement system in order to allocate hospital resources for children with special health care needs in a more equitable and affordable way [19].

We also identified independent predictors for becoming LSPs that are specific for each major diagnostic group. The impact of early readmission changes according to major diagnostic category. It has been already shown how a higher risk of mortality predicts higher LOS [4]. In our cohort of patients youngest age (<1 month) and higher PIM2 increase odds of becoming LSPs regardless of diagnostic category, although a higher risk of mortality at admission does not predict a diagnosis of LSPs for injured patients. Our data also show that the majority of patients that will eventually become LSPs have significantly higher demands of care at admission as expressed by higher NEMS. The knowledge of the aforementioned diagnostic-specific predictors might contribute to the timely identification of potential LSPs.

Based on our results, we believe that a more adapted approach based on actual age, diagnostic category and patients’ characteristics at PICU admission is needed when it comes to the definition and early identification of LSPs. We propose the systematic application of LSPs definition (i.e. patients who fell into the top 10^th^ percentile LOS distribution) for every major diagnostic and age category in order to define LSPs according to the actual clinical course of patients. This approach would also allow clinicians to apply LSPs definitions that reflect the real distribution of these patients referring to their PICU.

Our study has strengths. We emphasized issues pertinent to clinicians and patients rather than providing a-priori definition of LSPs. Moreover this study was done in a large, national database containing vast clinical information, ongoing audits of data quality and validation procedures.

Our study has limitations. One important limitation is its retrospective nature. Moreover the availability of support facilities like rehabilitation and social care services may have varied among PICU and could have had an impact on the outcome. This information was not available for our study. We could not explore the impact of several important patients’ characteristics on the occurrence of LSPs such as chronic or genetically influenced diseases [20]. Additionally, the ‘miscellaneous’ group resulted the most numerous group of patients. Given its heterogeneity, the generalizability of our results for this category of patients may be problematic. Besides diagnosis at admission, the MDSi database also collects associated diagnoses that comprise syndromes, congenital anomalies or diseases that are entered into the database if present at PICU admission or identified during admission. In our data it is not possible to distinguish several associated diagnoses from complications arising during PICU admission, as it is an inherent limitation to registry studies [21]. Therefore the impact of associated diagnoses was not explored in our study. This important distinction requires further prospective studies to determine the effect of concomitant underlying conditions on PICU LOS. Moreover, statistically significant but not clinically relevant results such as the difference in LSPs definition between genders, might have been the consequence of our large sample size instead of an underlying pathophysiological process.

## Conclusions

We found that predictors of LSPs change according to primary diagnosis. The use of a more adaptable definition of LSPs in PICU based on actual patients’ characteristics might potentially be able to improve care and preserve costs. An extended international study is warranted to confirm our conclusions in other PICU settings.
